# The Effect of Laser Biostimulation at Sensitized Acupoints on Chronic Pelvic Pain and Quality of Life in Women with Pelvic Inflammatory Disease: A Randomized Controlled Trial

**DOI:** 10.3390/medicina61020354

**Published:** 2025-02-18

**Authors:** Marwa Esmael Hasanin, Sobhy Mahmoud Aly, Mona Mohamed Taha, Lama Saad El-Din Mahmoud, Monira I. Aldhahi

**Affiliations:** 1 Department of Physical Therapy for Woman’s Health, Faculty of Physical Therapy, Cairo University, Cairo 12613, Egypt; marwa.esmael@pt.cu.edu.eg; 2 Physical Therapy for Women Health, Faculty of Physical Therapy, October University for Modern Sciences & Arts, Giza 12451, Egypt; 3 Department of Biomechanics, Faculty of Physical Therapy, Cairo University, Cairo 12613, Egypt; drsobhymahmoud@gmail.com; 4 Department of Medical Rehabilitation Sciences-Physiotherapy Program, College of Applied Medical Sciences, Najran University, Najran 55461, Saudi Arabia; 5 Department of Rehabilitation Sciences, College of Health and Rehabilitation Sciences, Princess Nourah bint Abdulrahman University, P.O. Box 84428, Riyadh 11671, Saudi Arabia; MOMTaha@pnu.edu.sa; 6 Department of Physical Therapy for Neurology and Neurosurgery, Faculty of Physical Therapy, October 6 University, Giza 12585, Egypt; lama.elsedawy.pt@o6u.edu.eg

**Keywords:** acupoints, laser b, quality of life, pain perception, pelvic pain, woman’s health

## Abstract

*Background and Objectives*: Chronic pelvic pain (CPP) is a common complication of pelvic inflammatory disease (PID). Although acupuncture has been increasingly used for the management of CPP, there is insufficient research to support the use of laser stimulation of sensitized acupoints. This study aimed to investigate the effects of laser on sensitized acupoints on pelvic pain perception and quality of life in women with pelvic inflammatory disease. *Materials and Methods*: Thirty-six women with CPP caused by chronic pelvic inflammatory disease were randomly divided into two equal groups. Both groups received non-steroidal anti-inflammatory drugs, while the study group also received a low-level laser at sensitized acupoints for 6 weeks. All the patients were evaluated before and after the intervention. The 12-item Short-Form Health Survey (SF-12) was used to measure the quality of life, while a pressure algometer and visual analog scale for pain (VAS-P) were used to measure pain sensitivity and intensity, respectively. A mixed MANOVA was used to investigate the effects of the treatment. *Results*: A mixed MANOVA on VAS, SF-12, and PPT scores revealed significant interaction effects of treatment and time (F = 38.34, *p* = 0.001, partial η^2^ = 0.96) and main effects of treatment (F = 9.74, *p* = 0.001) and time (F = 110.1, *p* = 0.001). Post-treatment, the study group showed greater reductions in VAS (MD = −1.00, *p* = 0.001), improvements in SF-12 (MD = 9.11, *p* = 0.001), and increased Pressure Pain Thresholds (PPT) at multiple anatomical points (*p* = 0.001–0.01). *Conclusions*: Laser biostimulation of neurogenic-sensitized acupoints significantly improves pain perception, intensity, and quality of life in women with CPP. suggested that the utilization of laser therapy at sensitized acupoints should be considered a potential component of a rehabilitation program for females suffering from chronic pelvic pain resulting from pelvic inflammatory disease. (Clinicaltrials.gov identifier: NCT05537207; Date of registration: 13 September 2022).

## 1. Introduction

Chronic pelvic pain (CPP) is a prevalent condition affecting numerous women globally, and is often a consequence of pelvic inflammatory disease (PID) [1]. The prevalence of PID has significantly increased, affecting approximately 4–12% of women of reproductive age worldwide [2]. Reports indicate that up to one-third of patients with a history of PID experience chronic pelvic pain, which is characterized as pain localized below the umbilicus and lasts for at least six months [3,4]. PID is an inflammatory condition of the female organs in the upper tract of the genitals and their surrounding structures induced by microbial infection, which frequently involves adjacent tissues [5]. Chronic pelvic inflammatory disease is a gynecological illness with complicated etiologies that are challenging to treat [1]. It has significant adverse effects on various aspects of a woman’s life, including social activities, mental and emotional health, job stability, and overall quality of life [6].

Traditional Chinese medicine (TCM), which has strong curative effects with few side effects, has received increasing attention in recent years. Acupuncture plays a significant role in the prevention and treatment of TCM [1]. Acupuncture increases stimulation by placing needles into meridian points and sending an electrical current through the acupoint, which makes it effective in reducing pain and is inexpensive, safe, and minimally invasive [7]. Compared to traditional acupuncture, laser acupoint biostimulation offers several advantages, including the ability to treat more acupoints with less discomfort and a reduced risk of infection, trauma, and bleeding problems [8]. Laser acupoint stimulation involves the application of low-power laser light to activate acupuncture points [9], utilizing non-thermal, low-intensity laser stimulation of the acupoints [10]. Low-level laser therapy (LLLT) offers an alternative to traditional acupuncture by delivering luminous energy to acupoints instead of using metal needles. This method induces photobiological effects encompassing biochemical, bioelectric, and bioenergetic responses. Initially, LLLT directly influences the epidermal neural network, subsequently extending its effects to the sympathetic ganglia, subcutaneous tissue nerves, and neuromuscular junctions within nerve trunks and muscles [11]. 

Previous studies confirmed the positive effects of acupoint stimulation on chronic pelvic pain. A study by Liu Ying-Hua et al. found that electroacupuncture combined with ibuprofen sustained-release capsules improved the symptoms, signs, and quality of life of patients with CPP compared to ibuprofen sustained-release capsules alone [12]. A previous study investigated the influence of electroacupuncture (EA) on chronic pelvic pain in individuals with a history of PID [13]. Visual analog scale (VAS) scores for pain in the lower abdomen and lumbosacral region decreased significantly after EA treatment [13]. Previous studies have investigated the impact of various types of sensitized acupoint stimulation, such as warm acupuncture [14], electroacupuncture [15], and dry needling [12,16]. However, limited research is available on the effects of laser treatment at sensitized acupoints on pain perception and quality of life in females caused by CPP. Therefore, the primary objective of this study was to examine the effects of laser application at sensitized acupoints on pain perception and quality of life in females with PID-induced chronic pelvic pain caused by PID.

## 2. Materials and Methods

A prospective, randomized, controlled trial was conducted using a pre- and post-experimental design. This study is approved by the Faculty of Physical Therapy at Cairo University in Egypt’s Institutional Ethics Committee approved the study (P.T. REC/012/003799), and it was prospectively registered on ClinicalTrials.gov with the identifier NCT05537207, prior to enrollment and randomization. The required sample size for the research groups was calculated a priori using GPower statistics (GPower version 3.1). A paired t-test was conducted with a significance level of 5%, a power of 80%, and an effect size of 0.98, using the Pain Rating Index data from Abdulaziz et al. [17], yielding a sample size of 18 participants per group [18].

Thirty-six married women diagnosed with chronic pelvic pain (CPP) and with a prior history of treatment for pelvic inflammatory disease (PID) were randomly selected from the Gynecological Outpatient Clinic at Cairo University Hospital, Egypt. This clinical trial included women who had periodic lower abdominal or pelvic pain for at least six months and met the clinical diagnostic criteria for PID based on the presence of all three major criteria: lower abdominal tenderness, cervical motion tenderness, and bilateral adnexal tenderness. Additionally, they had at least one minor supporting criterion: oral temperature >38.3 °C (101 °F), mucopurulent cervical discharge or cervical friability, abundant WBCs on saline microscopy of vaginal fluid, elevated ESR and/or CRP, or laboratory-confirmed cervical infection with N. gonorrhea or C. trachomatis [19]. The participants met the following requirements: they were between the ages of 25 and 40 years, had a maximum parity of three, had a body mass index (BMI) between 20 and 29.9 kg/m^2^, and had a VAS-P pain score of 4 cm or higher. The exclusion criteria included the presence of any other conditions causing pelvic pain (e.g., fibromyalgia, arthritis), a history of musculoskeletal or neurological diseases affecting the lower extremities and spine (such as lumbar disc herniation), acute pelvic inflammatory disease (PID), pregnancy or lactation, tumors, pelvic congestion, tuberculous PID, endometriosis, adenomyosis, gynecological tumors, or other gynecological conditions that could contribute to chronic pelvic pain (CPP). Additionally, participants with issues at acupoint sites (such as ulcers or skin conditions); impairments in other systems that could lead to pelvic and lumbosacral pain (e.g., digestive, motor, or urinary issues); severe systemic illnesses affecting the hematologic, digestive, urinary, or cardiovascular systems; mental illness; cognitive deterioration; immune-compromising diseases (such as diabetes or HIV/AIDS); and contraindications to NSAIDs were excluded.

Before starting the study, each participant read and signed the consent form. All procedures were performed in compliance with existing laws and institutional rules, which follow the principles outlined in the Declaration of Helsinki on the conduction of Human Research, and confidentiality and anonymity were assured.

### 2.1. Randomization and Allocation Assignment

Using a computer-generated, permuted block randomization process, participants were randomized into either the study or the control group. A random number generator with a 1:1 allocation ratio, six-block sizes, and random block size variation was used to create the randomization sequence. The study group received nonsteroidal anti-inflammatory drugs along with low-level laser therapy at sensitized acupoints, whereas the control group received only nonsteroidal anti-inflammatory medications.

**Figure 1:**
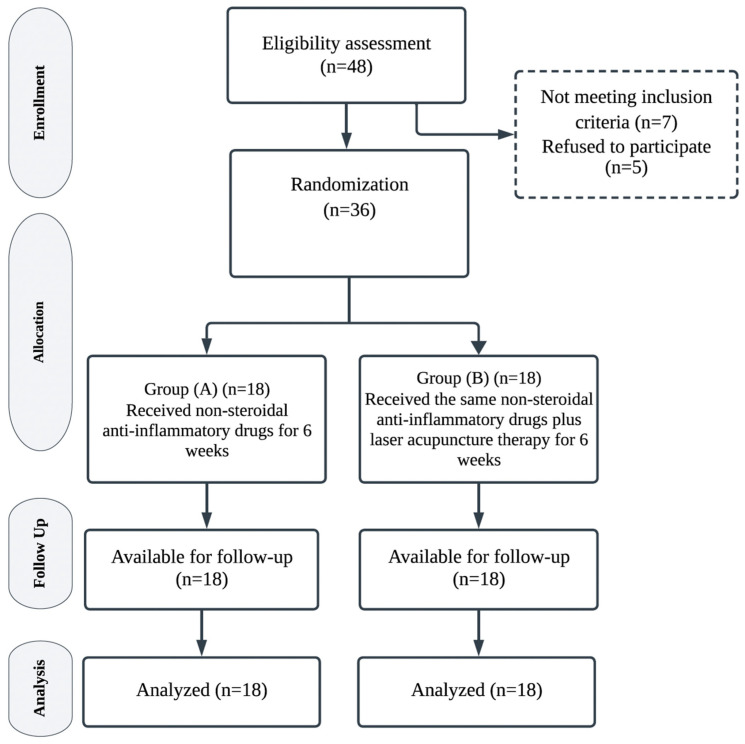
CONSORT study flow diagram.

The randomization list was created by a statistician who was not involved in participant enrollment or assessment to ensure the concealment of the allocation sequence. The allocation sequence was maintained in sequentially numbered opaque sealed envelopes. The participant’s group assignment was disclosed at the time of enrollment when the study coordinator opened the next numbered packet. The patients and assessors who completed the evaluations were double-blinded, meaning that they were not aware of the patients’ treatment group. The consolidated standards of reporting trials (CONSORT) flowchart of the study is shown in Figure 1.

### 2.2. Outcome Measures

#### 2.2.1. Pressure Pain Thresholds

Pain sensitivity was evaluated by measuring pressure pain thresholds (PPT) using the Pressure Algometer: A standardized 1.52 cm^2^ flat circular probe, the Baseline Dolorimeter, (New York 10533, NY, United States), was utilized to measure pain sensitivity by determining the PPT [20], and the selected measuring points included anterior 1st lumbar points (A1Ls; located medially to the anterior superior iliac spine), anterior 2nd lumbar points (A2Ls; located on the medial surface of the anterior inferior iliac spine), abdominal 2nd lumbar points (AB2Ls; located on the abdominal surface, approximately 5 cm laterally and somewhat caudally to the umbilicus, corresponding to the lateral margin of the rectus abdominis muscle), iliac points (ILs; located approximately 3 cm medial to the anterior superior iliac spine and deep into the iliac fossa), superior pubic points (SPs; located on the superior surface of the pubic area, approximately 2 cm lateral to the pubic symphysis), and posterior 5th lumbar points (P5L; on the posterior surface of the 5th transverse processes) [21].

The mean values of the right and left pressure-pain thresholds were computed for each site for analysis. Three measurements were made at each location with a ten-second interval between them.

#### 2.2.2. Quality of Life

A 12-Item Short-Form Health Survey (SF-12) was used to evaluate women’s quality of life in terms of physical functioning, role-physical functioning, general health, physical pain, vitality, role-emotional functioning, social functioning, and mental health. A higher overall score indicated a better health condition on a scale from 0 to 100 [22]. The SF-12 has been shown to be a valid and reliable test.

#### 2.2.3. Chronic Pelvic Pain Intensity

The Visual Analog Scale for Pain (VAS-P) was used to measure CPP intensity. On the VAS-P line, each woman was asked to mark the point between no pain and severe pain related to CPP severity. To calculate the CPP intensity score, centimeters were measured from the left end of the line to the detected point [23].

### 2.3. Interventions

According to the gynecologist’s instructions, both groups received non-steroidal anti-inflammatory drugs (NSAIDs) (ibuprofen 400 mg) twice a day after meals, for six weeks. The study group additionally received LLLT at sensitized acupoints for three sessions per week, for six weeks. LLLT was applied to the acupuncture points (BL33, BL35, SP6, SP9, LR3, and LI4) [24]. The patient was in a supine position at all times except for BL33 and BL35, which were in a prone position with a cushion under the abdomen. First, alcohol was used to clean the skin on the treated area. The therapist then indicated the position of each acupuncture point on the skin by using a skin marker. The laser device probe was held perpendicular to the acupuncture points in contact with the skin for 90 s throughout each session [25]. The following parameters were used in this study: wavelength, 904 nm; laser probe power density, 15 J/cm^2^; pulse repetition frequency, 5000 Hz; distance between the laser probe and skin, direct contact; and time, 90 s per point.

### 2.4. Statistical Analysis

The data were analyzed using IBM SPSS (version 25 for Windows, Chicago, IL, USA). To assess whether the data followed a normal distribution, the Shapiro-Wilk test was used. Levene’s test for the homogeneity of variances was used to assess the equality of variances between groups. An independent t-test was performed to compare baseline participant attributes between the groups, ensuring equivalence before treatment. To evaluate the effects of treatment on outcome measures, including the Visual Analog Scale (VAS), SF-12, and PPT scores, a mixed multivariate analysis of variance (MANOVA) was conducted to assess treatment effects across multiple dependent variables, while accounting for within- and between-group interactions. The Bonferroni correction was used to account for Type I error in 14 comparisons, yielding an adjusted significance level of α adjusted = 0.00357. While this conservative strategy reduces the risk of false positives, it also raises the likelihood of Type II errors, which may result in overlooking the true effects. The trade-off was evaluated, and both unadjusted (*p* < 0.05) and Bonferroni-adjusted *p*-values are presented to provide a full explanation of the results. The level of significance was set at *p* < 0.05.

## 3. Results

Table 1 presents a comparison of the baseline characteristics between the study and control groups. There were no significant differences between the groups in terms of age, weight, height, BMI, or duration of menstruation.

At baseline, there were no significant differences between the groups in VAS and QoL (*p* = 0.31, *p* = 0.27, respectively). Post-treatment, the study group showed a significantly greater reduction in VAS scores compared to the control group, with an MD of −1.00 (*p* = 0.001). Thus, there was a significantly greater improvement in SF-12 scores than in the control group, with an MD of 9.11 (*p* = 0.001).

A significant interaction effect of treatment and time was found using mixed MANOVA (F = 38.34, *p* = 0.001, partial eta-squared = 0.96). The main effect of the treatment was significant (F = 9.74, *p* = 0.001, partial eta-squared = 0.86). There was a significant main effect of time (F = 110.1, *p* = 0.001, partial eta-squared = 0.98). The percent change in VAS and SF-12 scores in the study group was 48.56% and 20.46%, respectively, whereas it was 32.61% and 11.48% in the control group (Table 2).

Table 3 shows the comparison of pressure pain at different anatomical points within and between the study and control groups before and after treatment. Within-group comparison revealed that there was a significant increase in Pressure Pain Thresholds (PPT) at the right and left A1Ls, A2Ls, and Ils acupoints (Table 3). Between-group differences revealed that the study group demonstrated a significant increase in PPT post-treatment which was statistically significant at several points, including RT A1Ls (MD: 2.09, *p* = 0.002), LT A1Ls (MD: 1.64, *p* = 0.01), RT A2Ls (MD: 1.3, *p* = 0.008), LT A2Ls (MD: 1.38, *p* = 0.009), RT Ils (MD: 2.6, *p* = 0.001), LT Ils (MD: 1.82, *p* = 0.005), RT AB2Ls (MD: 1.77, *p* = 0.001), LT AB2Ls (MD: 2.08, *p* = 0.001), RT Sps (MD: 0.34, *p* = 0.006), LT Sps (MD: 0.55, *p* = 0.001), RT P5L (MD: 0.55, *p* = 0.001), and LT P5L (MD: 0.66, 95% CI: 0.37 to 0.94, *p* = 0.001). The study does not report any adverse effects or complications arising for the patients during the study.

## 4. Discussion

The current study investigated the effect of low-level laser therapy at sensitized acupoints on pain perception and quality of life in adult females with chronic pelvic pain attributed to pelvic inflammatory disease. The findings demonstrated significant improvements in both visual analog scale (VAS) scores and health-related quality of life compared to the control group, supporting the effectiveness of laser treatment. Furthermore, the study group experienced significantly greater improvements in PPT at multiple anatomical points than the control group, which underscores the effectiveness of LLLT at sensitized acupoints.

These findings align with those reported by Sagir et al. [26], who observed significant improvements in VAS scores and PPT values following a 6-week course of LLLT targeting trigger points in the abdominopelvic region of females with CPP. Similarly, a recent meta-analysis corroborated the efficacy of laser acupuncture in alleviating musculoskeletal pain compared to sham therapy, as assessed using the VAS [27]. Baxter et al. [28] also provided moderate evidence for the clinical efficacy of laser acupuncture for myofascial pain syndrome. Collectively, these studies reinforce our findings, indicating that laser therapy can be beneficial for pain management in various clinical settings.

The concept of “acupoint sensitization” provides a theoretical basis for these findings. In traditional acupuncture therapy, “acupoint sensitization” refers to the natural state of an acupoint. Research has shown that the sensory threshold and biophysical properties of these acupoints can change in response to pathological stressors, such as diseases or injuries, which affect the body [29]. One of the most characteristic manifestations of hypersensitive acupoints is a lowered sensory threshold [29]. Stimulation of these sensitized acupoints elicits distinct neural responses that differ from the sensation of pain, reducing both affective and sensory inflammatory pain, as well as neuropathic pain. Additionally, combining acupoint stimulation with low-dose conventional analgesics offers an effective pain management strategy, minimizing the adverse effects associated with pharmaceutical treatments [30]. Therefore, the therapeutic effectiveness of laser acupuncture in alleviating pain may be linked to its capacity to modulate the flow of vital energy along the meridians of the body. Each meridian regulates particular bodily functions in connection with organs or organ systems, and the stimulation of acupoints is believed to harmonize this energy flow and promote overall health [27].

Trigger points and sensitized acupoints can be stimulated to elicit specific responses, such as twitch response and sensory transmission at the acupoint [31]. This specific stimulation has been shown to enhance treatment effectiveness. When applied at low doses, LLLT enhances the proliferation of lymphocytes, fibroblasts, keratinocytes, and endothelial cells. This is attributed to photostimulation of the mitochondria, which activates signaling pathways and upregulates transcription factors, leading to increased production of growth factors [32]. Unlike traditional needle acupuncture, non-thermal low-intensity laser irradiation minimizes risks such as bleeding, infection, and pain. The laser beam activates the acupoints through energy deposition without generating heat [9]. Furthermore, applying LLLT to peripheral nerves inhibits synaptic activity in second-order neurons, thereby preventing cortical activation of the pain matrix. It also regulates neurotransmitters, further supporting its pain-relieving effects [33]. External stimulation sensitizes nociceptors, leading to increased excitability, and a lowered activation threshold. Sensitive acupoints, especially those with active nociceptors such as C-nociceptors, generate stronger afferent impulses, contributing to their superior therapeutic effects [34]. Both A-delta and C nociceptors can be activated by acupuncture innervation depending on the intensity of stimulation, with C nociceptors playing a particularly significant role in acupoint sensitization [35].

This is further supported by Cui et al. [36], who demonstrated that peripheral nociceptors act as initial responders to acupoint stimuli. Whether activated by external factors, such as peripheral stimulation, or internal factors, such as tissue injury or visceral diseases, nociceptors generate action potentials in their sensory terminals. This heightened sensitivity, termed sensitization, reduces the threshold required for activation and increases responsiveness to stimuli, contributing to adaptive changes in sensitized acupoints [36]. Blocking nociceptors directly results in pain relief, as evidenced by conduction blockade in somatosensory-evoked potentials. Additionally, inhibiting peripheral sensitization reduces neuron activation thresholds and suppresses the synthesis of pro-inflammatory neuropeptides, such as substance P and calcitonin gene-related peptides [37].

Although women with chronic pelvic pain (CPP) often experience a lower quality of life, it remains uncertain whether pain relief directly improves mood and quality of life [38]. Given that the severity of pain can have a detrimental impact on quality of life [39,40], the current study observed improvements in activities of daily living (ADL) corresponding to the alleviation of CPP. Furthermore, our findings are consistent with those of Atas et al. [41], who demonstrated the efficacy of low-level laser therapeutic approaches, which can be safely utilized in CPP patients to improve their symptoms and related quality of life. However, no side effects of LLLT were observed in this study. It is important to mention that LLLT may lead to potential adverse effects, including burns, ocular injuries, dyspigmentation, scarring, acne, persistent erythema, milia, and contact dermatitis [42]. To mitigate the risk of these negative outcomes, it is crucial to implement appropriate precautions, such as precise control of the laser wavelength and pulse duration [42].

This study has several limitations that need to be acknowledged. A relatively small sample size, with only 36 participants, may limit the extrapolation of conclusions to a wider demographic. Although the sample size was determined to ensure sufficient statistical power to detect significant differences within the study parameters, increasing the sample size in future studies could improve the generalizability of the results. This would allow a more robust understanding of the effects of the intervention across diverse demographic and clinical groups. Additionally, the long-term effects of the intervention remain unclear, as the study was conducted within a six-week timeframe of low-level laser therapy. Future studies with larger sample sizes and longer follow-up durations are necessary to fully evaluate the sustained effects of these treatments over an extended period and the lasting effects of this intervention. Furthermore, controlling for confounding variables such as psychological well-being, lifestyle, and comorbidities is a limitation that should be considered in future studies. This study focused solely on females with CPP caused by PID, and the findings may not be applicable to males or females with other causes of CPP. Future research should consider gender disparities in understanding the efficacy of LLLT at sensitized acupoints in managing CPP. Although this study did not specifically address pelvic organ prolapse (POP), its relationship with PID may have an impact on symptoms and clinical outcomes. Future research should assess how these factors interact to better understand their combined impacts.

## 5. Conclusions

This study suggests that low-level laser therapy (LLLT) at sensitized acupoints can effectively reduce CPP and improve quality of life in women with PID Significant improvements were observed in pain perception, intensity, and overall health-related quality of life in the study group. These findings indicate that LLLT at sensitized acupoints may be a valuable addition to rehabilitation programs in women with CPP secondary to PID. However, further research with larger and more diverse samples, extended follow-up periods, and control for potential confounding factors is necessary to confirm these results and evaluate the long-term benefits of this therapy.

## Figures and Tables

**Table 1:** Comparison of baseline characteristics between study group and control group.

Variables	Study Group N = 18	Control Group N = 18	MD	t	*p*-Value
	Mean ± SD	Mean ± SD			
Age (years)	33.11 ± 6.71	35.50 ± 2.71	−2.39	−1.40	0.17
Weight (kg)	74.71 ± 7.77	75.17 ± 4.33	−0.46	−0.21	0.83
Height (cm)	165.06 ± 9.84	166.22 ± 7.95	−1.16	−0.39	0.69
BMI (kg/m^2^)	27.41 ± 1.21	27.28 ± 1.94	0.13	0.22	0.82
Duration of menstruation (days)	6.11 ± 2.08	5.88 ± 1.52	0.23	0.36	0.71

Abbreviations: BMI, body mass index; SD, standard deviation; MD, mean difference.

**Table 2:** Comparison of the VAS and SF-12 score within and between groups pre- and post-treatment.

Variables	Pre-Treatment N = 18	Post-Treatment N = 18	MD (95%CI)	*p* Value
	Mean ± SD	Mean ± SD		
VAS sore				
Study group	7.66 ± 0.91	3.94 ± 0.8	3.72 (3.2–4.24)	0.001 **
Control group	7.33 ± 1.02	4.94 ± 0.87	2.39 (1.72–3.06)	0.001 **
MD (95%CI)	0.33 (−0.32–0.99)	−1 (−1.57–−0.43)		
*p* value	0.31	0.001 **		
SF-12 score				
Study group	71.16 ± 6.16	85.72 ± 5.92	−14.56 (−16.95–−12.15)	0.001 **
Control group	68.72 ± 6.98	76.61 ± 7.2	−7.89 (−10.28–−5.49)	0.001 **
MD (95%CI)	2.44 (−2.01–6.91)	9.11 (4.64–13.58)		
*p* value	0.27	0.001 **		

Abbreviations: AS, visual analog scale; SF−12, 12-item Short-Form Health Survey; SD, Standard deviation; MD, Mean difference; CI, Confidence interval, ** denotes *p* < 0.004 (Bonferroni-corrected significance level).

**Table 3:** Comparison of the pressure pain thresholds (PPT) at different anatomical points within and between groups pre- and post-treatment.

PPT	Groups	Pre-Treatment	Post-Treatment	MD (95%CI)	*p* Value
		Mean ± SD	Mean ± SD		
RT A1Ls	Study group	3.95 ± 2.01	5.75 ± 1.73	−1.8 (−1.98–−1.63)	0.001
	Control group	3.48 ± 1.91	3.66 ± 1.94	−0.18 (−0.36–−0.01)	0.03
	MD (95%CI)	0.47 (−0.85–1.8)	2.09 (0.84–3.33)		
	*p* value	0.47	0.002 **		
LT A1Ls	Study group	3.73 ± 2.07	5.29 ± 1.82	−1.56 (−1.85–−1.27)	0.001
	Control group	3.29 ± 1.91	3.65 ± 1.96	−0.36 (−0.64–−0.06)	0.01
	MD (95%CI)	0.44 (−0.91–1.78)	1.64 (0.36–2.93)		
	*p* value	0.51	0.01 *		
RT A2Ls	Study group	3.67 ± 1.39	5.31 ± 1.48	−1.64 (−1.93–−1.34)	0.001
	Control group	3.61 ± 1.21	4.01 ± 1.28	−0.4 (−0.69–−0.1)	0.01
	MD (95%CI)	0.06 (−0.82–0.94)	1.3 (0.35–2.23)		
	*p* value	0.89	0.008 *		
LT A2Ls	Study group	3.58 ± 1.31	5.53 ± 1.48	−1.95 (−2.18–−1.71)	0.001
	Control group	3.55 ± 1.65	4.15 ± 1.52	−0.6 (−0.83–−0.36)	0.001
	MD (95%CI)	0.03 (−0.97–1.04)	1.38 (0.36–2.41)		
	*p* value	0.94	0.009 *		
RT Ils	Study group	3.16 ± 1.04	6.68 ± 0.91	−3.52 (−3.87–−3.16)	0.001
	Control group	3.38 ± 1.06	4.08 ± 1.16	−0.7 (−1.05–−0.35)	0.001
	MD (95%CI)	−0.22 (−0.93–0.49)	2.6 (1.88–3.31)		
	*p* value	0.53	0.001 **		
LT Ils	Study group	3.28 ± 1.59	6.04 ± 1.69	−2.76 (−3.45–−2.06)	0.001
	Control group	3.18 ± 1.53	4.22 ± 1.97	−1.04 (−1.74–−0.34)	0.005
	MD (95%CI)	0.1 (−0.95–1.16)	1.82 (0.57–3.06)		
	*p* value	0.84	0.005 *		
RT AB2Ls	Study group	4.26 ± 1.95	6.75 ± 1.34	−2.49 (−2.93–−2.05)	0.001
	Control group	4.22 ± 1.67	4.98 ± 1.48	−0.76 (−1.19–−0.31)	0.001
	MD (95%CI)	0.04 (−1.19–1.26)	1.77 (0.81–2.72)		
	*p* value	0.95	0.001 **		
LT AB2Ls	Study group	4.12 ± 1.08	6.71 ± 1.53	−2.59 (−2.97–−2.17)	0.001
	Control group	4.08 ± 1.23	4.63 ± 1.17	−0.55 (−0.95–−0.15)	0.009
	MD (95%CI)	0.04 (−0.74–0.83)	2.08 (1.13–2.99)		
	*p* value	0.91	0.001 **		
RT Sps	Study group	0.82 ± 0.33	1.36 ± 0.39	−0.54 (−0.65–−0.43)	0.001
	Control group	0.89 ± 0.27	1.02 ± 0.30	−0.13 (−0.24–−0.02)	0.01
	MD (95%CI)	−0.07 (−0.27–0.14)	0.34 (0.11–0.57)		
	*p* value	0.52	0.006 *		
LT Sps	Study group	0.73 ± 0.21	1.47 ± 0.22	−0.74 (−0.84–−0.63)	0.001
	Control group	0.77 ± 0.16	0.92 ± 0.18	−0.15 (−0.26–−0.05)	0.005
	MD (95%CI)	−0.04 (−0.16–0.09)	0.55 (0.41– 0.68)		
	*p* value	0.58	0.001 **		
RT P5L	Study group	1.26 ± 0.59	2.05 ± 0.43	−0.79 (−0.93–−0.63)	0.001
	Control group	1.30 ± 0.48	1.51 ± 0.41	−0.21 (−0.36–−0.06)	0.009
	MD (95%CI)	−0.04 (−0.40–0.33)	0.55 (0.25–0.82)		
	*p* value	0.84	0.001 **		
LT P5L	Study group	1.34 ± 0.39	2.20 ± 0.43	−0.86 (−1.03–−0.69)	0.001
	Control group	1.25 ± 0.33	1.54 ± 0.39	−0.29 (−0.46–−0.12)	0.001
	MD (95%CI)	0.09 (−0.15–0.33)	0.66 (0.37–0.94)		
	*p* value	0.46	0.001 **		

Abbreviations: PPT, pressure pain thresholds; A1Ls, anterior 1st lumbar points; A2Ls, anterior 2nd lumbar points; Ils, iliac points; RT, right; LT, left; SD, Standard deviation; MD, Mean difference; CI, Confidence interval; AB2Ls, abdominal 2nd lumbar points; Sps, superior pubic points; P5L, posterior 5th lumbar points; RT, right; LT, left; SD, Standard deviation; MD, Mean difference; CI, Confidence interval, * *p* < 0.05 (uncorrected significance level), ** p < 0.004 (Bonferroni-corrected significance level).

## Data Availability

The data are not publicly available because further research is being conducted and more manuscripts are being prepared. Data for the current study will be available upon reasonable request from the principal investigator or corresponding author.
